# Predictors of incident heart failure in a cohort of patients with ischemic heart disease

**DOI:** 10.11604/pamj.2020.35.45.18257

**Published:** 2020-02-17

**Authors:** Senbeta Guteta Abdissa

**Affiliations:** 1 Division of Cardiology, Department of Internal Medicine, School of Medicine, College of Health Sciences, Addis Ababa University, Ethiopia

**Keywords:** Incidence, heart failure, ischemic heart disease, predictors

## Abstract

**Introduction:**

heart failure (HF) is a major complication following ischemic heart disease (IHD) and it adversely affects the outcome. The objective of this study was to identify predictors of HF in patients with IHD.

**Methods:**

this is a 24-month longitudinal retrospective study of all consecutive patients diagnosed with IHD. Endpoints were incident HF and time to incident HF. Patients with a previous history of HF were excluded.

**Results:**

a total of 306 patients with IHD were included in the analysis. The 6-month, 12-month and 18-month cumulative risk of developing incident HF were 18.8%, 28.4%, and 53.5% respectively. Increasing age, female gender, diabetes mellitus (DM), lower hemoglobin, and dilated left atrium were strong predictors of incident HF. Predictors of shorter time to incident HF were coexisting DM and hypertension, and the presence of dilated left atrium in patients with left ventricular ejection fraction < 40%. The strongest predictor of incident HF in patients with DM was a higher level of LDL cholesterol.

**Conclusion:**

patients with IHD have a higher risk of incident HF. Strong predictors of incident HF in these patients were increasing age, female gender, DM, lower hemoglobin and dilated left atrium. Such patients need close follow-up and more intensive treatment.

## Introduction

Ischemic heart disease (IHD) is one of the major underlying causes of heart failure (HF) [1–3]. In sub-Saharan Africa, IHD was formerly considered rare, but reports from 2008 indicate that it ranks 8th among the adult leading causes of death in the region. IHD in Ethiopia has also dramatically increased over the last 30 years, from 88 to 960 per 100,000 patients with cardiovascular disease [4–7]. IHD increases the risk of HF by about 8 times, with a population attributable risk of 65% in men and 48% in women [8]. HF is a clinical syndrome characterized by symptoms of breathlessness, ankle swelling, and fatigue that may be accompanied by signs such as elevated jugular venous pressure, pulmonary crackles and peripheral edema [9]. Although the growing use of coronary artery revascularization procedures and better medical treatment have largely improved the outcome of an acute coronary syndrome, heart failure as a complication of IHD still remains a problem because of a scarcity of resources for proper intervention, particularly in resource-limited settings. Even in settings with coronary revascularization, IHD still remains one of the leading causes of clinical HF onset. The possible mechanisms include loss of functioning myocytes, fibrosis of myocardium, and subsequent remodeling of left ventricular (LV) that adversely affects LV function [10]. Furthermore, the risk of HF may increase from chronic myocardial dysfunction resulting from hypoperfusion/hibernation [11,12]. A clear knowledge of the risk factors or predictors of HF after IHD could guide therapeutic decisions and monitoring by identifying high-risk patients and pursuing more strict treatment and close follow-up in certain subsets. This study aimed to identify predictors of incident HF in a cohort of patients with IHD.

## Methods

**Study design and clinical setting**: this retrospective cohort study was designed to assess all consecutive patients with a diagnosis of IHD coming to Black Lion Specialized and tertiary referral Hospital in Addis Ababa, Ethiopia. The study population was consecutively recruited and enrolled starting from November 30, 2015. A total of 306 adults (age of 18 years and above) IHD patients were included in the cohort and they were followed to measure the outcomes. Each study participant was followed up for 24 months or until the diagnosis of HF was made. Patients with IHD or HF were identified based on the treating Physician’s final diagnosis that was made based on symptoms suggestive of IHD or HF. The study was approved by the institutional review board of the College of Health Sciences, Black Lion Hospital, and permission to use de-identified personal healthcare information for all included subjects was obtained.

**Variables definition**: patients with preceding IHD were identified by searching for those who already had a clinical diagnosis of MI, and/or a history of angina or angina-driven coronary revascularization. Preceding HF was identified if patients had HF diagnosis or if they had typical signs and symptoms consistent with a HF syndrome and/or patient with the use of furosemide as part of their treatment, along with compatible imaging studies (echocardiogram). Patients who were diagnosed with HF or used furosemide during the follow-up period were categorized as having incident HF. Those who did not have the diagnosis of HF throughout the study period were categorized into the group with no incident HF.

**Inclusion and exclusion criteria**: IHD patients with who were in follow up until the study exit date, or developed HF, or died, were included. The exclusion criteria included IHD patients who were diagnosed to have HF or were using furosemide before the date of enrollment. Those who were lost to follow up before the study exit date, diagnosed with an end-stage renal disease or severe liver disease were also excluded.

**Data Collection**: data on baseline characteristics, risk factors, comorbidities, diagnosis and date of diagnosis of IHD and HF, functional classification of HF based on New York Heart Association (NYHA), complementary laboratory tests were collected from the medical records by trained medical staff. Resting 12-lead electrocardiographs, transthoracic echocardiography findings, medications and hospital admission were collected from the patient’s medical records. New onset HF was defined if the patient was diagnosed as having HF as principal or secondary diagnosis or used furosemide during any time of the follow-up period. Based on echocardiographic LVEF data patients were categorized into groups depending on the diagnosis of HF and LVEF classification as reduced (<40%), mid-range (40-49%) and normal (≥ 50%). The study design is an observational cohort study with retrospectively collected data. The Institutional Review Board of the College of Health Sciences at Addis Ababa University approved this study.

**Outcome measures**: the primary outcome of interest was incident HF. The calculated power of the study to detect a difference was 93.7%.

**Statistical analysis**: baseline characteristics by incident HF were presented as numbers and percentages for categorical variables. Continuous parametric variables were expressed as means (± standard deviation). Categorical data were compared using Pearson’s Chi-square test while continuous data were compared using independent student t-test. The diagnosis of incident HF was taken as a poor prognostic factor. Factors predicting the risk of incident HF were explored in a univariate and multivariate Cox-regression model with incident HF as the outcome (dependent) variable and all covariates described below as predictor (independent) variables. In the multivariate model, all variables associated with the evaluated endpoint at the 0.10 level in the univariate analysis were entered in the model and removed using a backward stepwise likely-hood ratio selection process for determination of predictors of late-onset HF. For each covariate, HR, 95% CI and p-value are reported. Covariates included in the multivariable analysis were age, gender, comorbidities including diabetes mellitus (DM), hypertension (HTN), dyslipidemia, LVEF, and left atrial dimension. The cumulative probability of incident HF and time to incident HF was illustrated with Kaplan-Meier time to event curve estimates. Cox regression analysis was used to calculate the hazard ratios for predictors of HF. In cases of missing data, the “*Exclude cases pair wise*” option was used during analysis. A 5% significance level was adopted for all tests and all tests were 2-sided. Statistical analyses were performed using SPSS 23.0 (IBM).

## Results

**Figure 1:**
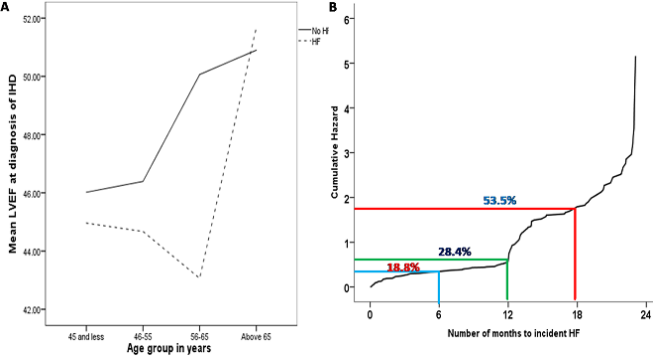
A) Line graph of the LVEF of IHD patients with and without HF across the four age groups; B) Kaplan Meier plot showing the risk of incident HF over 24-month period for an index IHD between 2015 and 2017

**Baseline characteristics**: out of 306 IHD patients without prior HF before November 2015, 64.1% (n= 196) developed HF during the study period, with a follow up of 24 months. Patients who developed HF were older, more often female, diabetic, had lower hemoglobin and bigger left atrium. The proportion of patients taking calcium channel blockers and antidiabetic medications was higher in those who developed HF during follow up compared to those who did not develop HF (Table 1). Comorbidities and risk factors such as hypertension, dyslipidemia, smoking, cerebrovascular accidents, and peripheral arterial diseases were similar between the two groups. There was no significant difference in baseline left ventricular dimensions, nephropathy and blood pressure parameters between those who developed HF and those who did not develop HF. Although it did not reach statistical significance, patients with IHD who developed HF appear to have a lower level of baseline left ventricular ejection fraction (LVEF) than those who did not develop HF (Figure 1 A). In both patient groups, LVEF appears to be higher in older patients. The 6-month, 12-month and 18-month cumulative risk of developing HF were 18.8%, 28.4%, and 53.5% respectively (Figure 1 B).

**Table 1:** Baseline characteristics of patients with and without incident HF

Variables	All patients (N=306)	Incident HF (N=196, 64.1%)	No incident HF (N=110, 35.9%)	P-value
**Baseline age (years)**				
45 and less (n, %)	59 (19.3)	29 (14.8)	30 (27.3)	1.00
46-55 (n, %)	96 (31.4)	62 (31.6)	34 (30.9)	0.019
56-65	88 (28.8)	61 (31.1)	27 (24.5)	0.007
66 and above	63 (20.6)	44 (22.4)	19 (17.3)	0.004
Mean (±SD) age	56.8 (11.6)	57.9 (11.1)	54.5(12.2)	0.014
**Gender**				
Male (%)	211 (69.0)	124 (63.3)	87 (79.1)	0.003
Female (%)	95 (31.0)	72 (36.7)	23 (20.9)	1.00
**Comorbidity**				
HTN	144 (47.1)	98 (50.0)	46 (41.8)	0.17
DM	96 (31.4)	70 (35.7)	26 (23.6)	0.029
Dyslipidemia	48 (15.7)	31 (15.8)	17 (15.5)	0.93
Cerebrovascular accident	15 (4.9)	12 (6.1)	3 (2.7)	0.19
Peripheral arterial disease	3 (1.0)	2 (1.0)	1 (0.9)	0.92
**Smoking status**				
Never smoked (%)	258 (84.3)	167 (85.2)	91 (82.7)	1.00
Ever/Current smoker (%)	48 (15.7)	29 (14.8)	19 (17.3)	0.48
**Admission to hospital**				
Yes (%)	80 (26.1)	141 (71.9)	85 (77.3)	0.22
No (%)	226 (73.9)	55 (28.1)	25 (22.7)	1.00
**Blood Pressure**				
SBP (mmHg)-baseline	129.38	128.80	130.44	0.49
DBP (mmHg)-baseline	80.25	79.96	80.78	0.49
**Laboratory Data** mean or n (%)				
LVEF	46.30 (13.67)	45.65 (13.55)	47.65 (12.99)	0.29
LVDd (mm)	51.24 (10.30)	51.87 (10.96)	50.06 (8.89)	0.24
LAD (mm)	36.43 (6.75)	37.43 (6.76)	34.60 (6.39)	0.05
Hemoglobin (g/dl)	14.42 (1.97)	14.21 (1.98)	14.83 (1.89)	0.02
WBC count (mm3)	7943.93(2848.26)	7980.37 (2726.52)	7873.37 (3087.45)	0.79
Nephropathy (%)	103 (33.7)	62 (31.6)	41 (37.3)	0.32
**Medications (%)**				
Aspirin	281 (91.8)	181 (92.3)	100 (90.9)	0.66
Statins	261 (85.3)	164 (83.7)	97 (88.2)	0.29
B-blockers	254 (83.0)	168 (85.7)	86 (78.2)	0.09
RAS inhibitors	252 (82.4)	165 (84.2)	87 (79.1)	0.26
Clopidogrel	32 (10.5)	16 (8.2)	16 (14.5)	0.08
Hydrochlorthiazide	42 (13.7)	25 (12.8)	17 (15.5)	0.51
Calcium channel blocker	34 (11.1)	16 (8.2)	18 (16.4)	0.029
Antidiabetic medication	96 (31.4)	70 (35.7)	26 (23.6)	0.029
Insulin	24 (7.8)	19 (9.7)	5 (4.5)	0.11
Metformin	85 (27.8)	60 (30.6)	25 (22.7)	0.14
Sulfonylurea	12 (3.9)	10 (5.1)	2 (1.8)	0.16

Abbreviations: HF, heart failure; HTN, hypertension; DM, diabetes mellitus; SBP, systolic blood pressure; DBP, diastolic blood pressure; LVEF, left ventricular ejection fraction; LVDd, diastolic Left Ventricular dimension; LAD, left atrial dimension; WBC, white blood cell count

**Incident HF and its predictors**: increasing age, female gender, DM, lower baseline hemoglobin, LVEF less than 40% and bigger LAD increased the risk of incident HF on bivariate analysis (Table 2). After adjustment, age 66 years and above, female gender, DM, and bigger LAD were associated with an increased risk of incident HF. Age 66 years and above, and DM increased the risk of incident HF by about four-fold.

**Table 2:** Predictors of incident HF (bivariate and multivariate analysis)

Variables	COR*	95% CI	AOR**	95% CI
**Baseline age (years)**				
45 and less (n, %)	1.00		1.00	
46-55 (n, %)	1.89	0.98-3.65	2.08	0.74-5.89
56-65	2.34	1.18-4.63	2.56	0.85-7.67
66 and above	2.40	1.14-5.03	4.18	1.15-15.25
**Gender**				
Male (%)	1.00		1.00	
Female (%)	2.20	1.28-3.78	2.84	1.16-6.97
**DM**				
Yes (%)	1.80	1.06-3.04	3.67	1.49-9.05
No (%)	1.00		1.00	
**HTN**				
Yes (%)	1.39	0.87-2.23	1.32	0.56-3.11
No (%)	1.00		1.00	
**LVEF < 40%**				
Yes (%)	1.88	1.01-3.51	1.82	0.77-4.31
No (%)	1.00		1.00	
**LA**	t(196)=-2.87, two tailed		1.11	1.04-1.19
Hemoglobin	t(232)=2.30, two tailed		0.90	0.73-1.11

**Abbreviations:** HF, heart failure; HTN, hypertension; DM, diabetes mellitus; LVEF, left ventricular ejection fraction; LVDd, diastolic Left Ventricular dimension; LAD, left atrial dimension;

* COR= Crude Odds Ration,

** AOR= Adjusted Odds Ration CI=Confidence Interval

**Figure 2:**
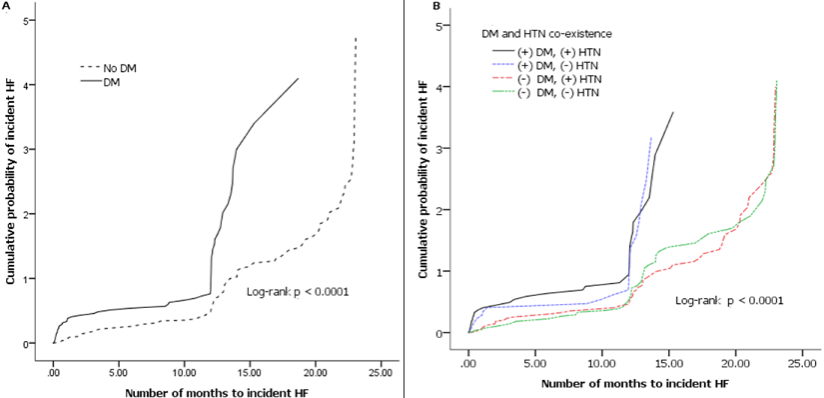
A) Kaplan-Meier curves for incident heart failure stratified by DM; B) Kaplan-Meier curves and Cox Hazard model for incident heart failure stratified by DM and HTN

**Predictors of incident HF by DM status**: patients with DM who developed HF were predominantly male and had a significantly higher proportion of advanced HF symptoms (functional class 3-4) and dislipidemia on bivariate analysis when compared to patients with incident HF but no DM. The time to incident HF was also significantly shorter in diabetic patients (Table 3). After adjustment, DM was associated with advanced HF symptoms and dyslipidemia. DM increased the risk of advanced HF symptoms by three-fold and the proportion of dyslipidemia was higher by about six-fold in patients with DM and HF compared to those HF patients without DM. The Kaplan-Meier procedure was used to estimate the incident HF curves (Figure 1). As compared with the non-diabetic IHD patients, the diabetic IHD patients had a significantly poorer prognosis [Log Rank (Mantel-Cox) [Chi- Square 28.4, df 1, p< 0.0001]]. The time to incident HF was also significantly shorter (7.8 months versus 12.4 months) in the diabetic IHD patients (Figure 2 A). When the patients were stratified according to DM and hypertension, non-diabetic IHD patients had the best prognosis (Figure 2 B) [Log Rank (Mantel-Cox) [Chi- Square 28.8, df 3, p< 0.0001]]. Compared with non-diabetic, non-hypertensive IHD patients (reference), diabetic-hypertensive IHD patients had a worse prognosis with HR of 2.57 (95% CI: 1.66-3.98, p <0.0001) and shorter time to incident HF (7.2 versus 12.4 months).

**Table 3:** Predictors of incident HF in IHD patients with and without DM (bivariate and multivariate analysis)

Variables	COR*	95% CI	AOR**	95% CI
**Baseline age (years)**				
45 and less (n, %)	1.00		1.00	
46-55 (n, %)	0.51	0.21-1.26	0.46	0.13-1.57
56-65 (n, %)	0.52	0.21-1.29	0.40	0.12-1.38
66 and above (n, %)	0.61	0.24-1.59	0.37	0.09-1.51
**Gender**				
Female (%)	1.00		1.00	
Male (%)	1.76	0.94-3.29	1.34	0.54-3.32
**NYHA class 3-4 HF (%)**				
No (%)	1.00		1.00	
Yes (%)	2.40	1.32-4.36	3.04	1.33-6.95
**HTN**				
No (%)	1.00		1.00	
Yes (%)	1.7	0.95-3.10	1.45	0.55-3.84
**LVEF**				
> = 50 (%)	1.00		1.00	
40-49(%)	0.87	0.36-2.12	0.81	0.25 – 2.56
< 40(%)	1.45	0.71-3.13	1.42	0.53-3.82
**LA**				
Normal	1.00		1.00	
Dilated	0.84	0.39-1.81	0.67	0.28-1.69
**Dyslipidemia**				
No (%)	1.00		1.00	
Yes (%)	5.96	2.56-13.87	5.96	2.56-13.87

**Abbreviations:** LVEF, left ventricular ejection fraction; LVDd, diastolic Left Ventricular dimension; LAD, left atrial dimension; WBC, white blood cell count;

* COR=Crude Odds Ration,

* AOR= Adjusted Odds Ration, CI=Confidence Interval

**Incidence and predictors of HF in diabetic IHD patients**: the incidence of HF was higher in patients with DM 72.9%, compared to patients with no DM 27.1%. The presence of DM was significantly associated with more admission to hospital, dilated LAD and higher levels of total cholesterol and LDL cholesterol (Table 4). On multivariate analysis, we find that the covariates contribute significantly to explaining the new onset HF [HF [X^2^ (11) = 23.68, p = 0.014]. After adjustment, DM was associated with higher levels of LDL cholesterol.

**Table 4:** Predictors of incident HF in diabetic IHD patients (bivariate and multivariate analysis)

Variables	COR*	95% CI	AOR	95% CI
**Baseline age (years)**				
45 and less (n, %)	1.00		1.00	
46-55 (n, %)	1.67	0.46-6.03	0.58	0.02-13.97
56-65 (n, %)	1.43	0.41-4.99	1.00	0.03-31.40
66 and above (n, %)	1.33	0.36-4.92	2.84	0.06-138.96
**Gender**				
Male (%)	1.00		1.00	
Female (%)	3.07	0.83-11.37	2.94	0.49-17.63
**Admission to hospital**				
Yes (%)	3.75	1.02-13.80	0.96	0.06-16.48
No (%)	1.00		1.00	
**Hypertension (%)**				
Yes (%)	1.65	0.67-4.08	2.17	0.72-6.25
No (%)	1.00		1.00	
**LAD (mm)**	t (63)= - 2.98, p = 0.004		1.20	0.94-1.53
Total cholesterol	t (69)= -3.09, p = 0.003		0.99	0.95-1.03
HDL	t(64)= - 0.49, p = 0.63		1.05	0.98-1.13
LDL	t(64)= - 2.72, p = 0.008		1.07	1.03-1.14
TG	t(67)= - 1.01, p = 0.32		0.99	0.97-1.07

* COR=Crude Odds Ration;

* AOR=Adjusted Odds Ration; CI=Confidence Interval; $:t-tests are two-tailed

**Figure 3:**
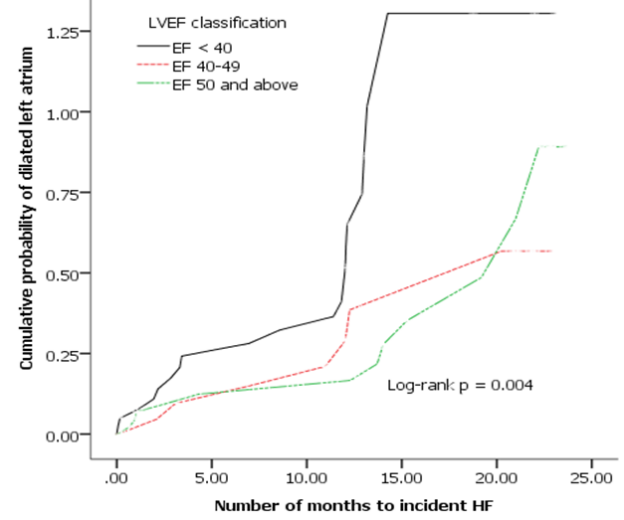
Kaplan Meier plot illustrating time to incident HF for the different LVEF categories in patients with dilated LA: LVEF < 40% (12.9, 95% CI 10.1-15.8); LVEF 40-49% (18.0, 95% CI 4.9-21.2), LVEF 50% and above (18.6, 95% CI 16.2-21.0)

**Predictors of HF in IHD patients by LVEF categories**: the mean left atrium (LA) dimension was bigger in those with lower LVEF and it decreased with increasing LVEF. The mean LA dimension was also larger in those patients with heart failure across the whole spectrum of LVEF categories. As shown in the Kaplan Meier plot (Figure 3), in IHD patients with dilated left atrium the time to incident heart failure was significantly shorter in the category of LVEF < 40% (12.9 months) compared to those with mid-range EF (18.0 months) and normal EF (18.6 months).

## Discussion

The findings of this longitudinal study for the duration of 24 months in patients with IHD indicate that incident HF is high. The risk of incident HF increases with increasing age, female gender, DM, lower hemoglobin, lower LVEF, and bigger LA. Predictors of shorter time to incident HF were a coexistence of DM with HTN, and coexistence of dilated LA with LVEF < 40%. The strongest predictor of incident HF in patients with DM was a higher level of LDL cholesterol.

**Predictors of incident HF**: the incidence of HF is larger than reports from the other studies [13,14]. The main reason could be because of lack of reperfusion practice for patients with acute myocardial infarction and late presentation of patients to the clinical setting. The other possible reasons are differences in characteristics of included patients, as well as differences in follow-up duration. Patients with DM or age above 66 years were 4 times as likely to develop incident HF. These risk factors are similar to those reported by other studies and are aligned with the present understanding of the pathogenesis of HF following IHD [15–17]. We also identified female gender and lower hemoglobin as parameters associated with the future risk of incident HF. These have also been shown in other studies. Anemia is clearly associated with increased adverse events including short-term mortality following IHD [13,18,19]. Furthermore, anemia is common in HF patients due to inflammation, iron deficiency, kidney disease, and use of ACE inhibitors [20]. LVEF less than 40% is also the other predictor for incident HF, similar to the other studies [21]. The other strong predictor of incident HF identified in this study was bigger LA.

**Predictors of incident HF by DM status**: patients with DM who developed HF were predominantly male, had a shorter time to incident HF, more admission to hospital and a three-fold higher risk of advanced HF symptoms. Dilated LA and HTN were additional predictors for incident HF and shorter time to HF in patients with DM. Reports from other studies also indicate poor prognosis in those patients with DM and other risk factors [22]. For incident HF, the strongest predictor of all the risk factors in diabetic patients in this study was a higher level of LDL cholesterol. These findings emphasize the importance of identifying and monitoring these risk factors for a better outcome in patients with DM.

**Predictors of HF in IHD patients by LVEF categories**: the mean left atrium (LA) dimension increased with decreasing LVEF. Across the whole spectrum of LVEF categories, mean LA dimension was larger in those patients who later developed HF. Moreover, time to incident HF was significantly shorter when low EF and dilated LA coexisted. The presence of low LVEF has an adverse LV remodeling following MI subsequently leading to heart failure [23]. Left atrium size gives additional information in identifying those patients with a high risk of developing HF in the future.

**Limitations and strengths**: This study has some limitations. Unmeasured confounders or details about a physician or patient decision-making that are not captured by the registry may have accounted for differences in treatments and outcomes. As in any observational study, we cannot rule out the effect of residual confounding due to unmeasured variables. Additionally, preceding HF was identified on a clinical basis but was different from the definition used for the primary endpoint defined appropriately by Framingham criteria. Even though Framingham criteria were not explicitly used by Doctors to determine preceding HF, it is the primary classification employed in our daily practice for the diagnosis of HF. As such, we do not believe that this difference is likely to affect our study results or conclusions. This study has strengths. We examined the role of multiple risk factors including left atrial size and lipid parameters on post-IHD prognosis including subgroups of DM and LVEF.

## Conclusion

Many clinical co-morbidities determine the risk for development of HF following IHD. Importantly, older age, gender, DM, left atrial size and LVEF were predictors of incident HF in patients with IHD. In patients with DM, the strongest predictors of incident HF were coexistent HTN and higher LDL cholesterol. While in patients with LVEF < 40%, the presence of dilated LA was a predictor of short time to incident HF. These findings are vital in assisting the clinician and researcher to identify patients at risk of developing HF. It is also an input for further studies and policy decision making. Patients with risk factors, if identified, may benefit from a close follow-up, more intensive medical treatment and additional interventions including effective intervention strategies such as percutaneous coronary intervention, and coronary artery bypass graft.

### What is known about this topic

- Heart failure remains a frequent complication of ischemic heart disease despite the use of revascularization in patients with acute coronary syndrome;
- Patients with diabetes have a two- to fourfold higher risk of HF than people without diabetes;
- The link between diabetes and the development of HF in patients with ischemic heart disease is not fully known.

### What this study adds

- The incidence of HF in Ethiopian patients with ischemic heart disease is larger than reports from the other studies;
- Dilated Left atrium and hypertension at baseline were predictors of incident HF and shorter time to HF in patients with diabetic ischemic heart disease compared to the non-diabetic counterparts;
- For incident HF, the strongest predictor of all the risk factors in diabetic IHD patients was a higher level of LDL cholesterol.

## Competing interests

The author declares no competing interests.
